# Isolation and Purification of *Drosophila* Peripheral Neurons by Magnetic Bead Sorting

**DOI:** 10.3791/1599

**Published:** 2009-12-01

**Authors:** Eswar Prasad R. Iyer, Srividya Chandramouli Iyer, Mikolaj J. Sulkowski, Daniel N. Cox

**Affiliations:** Department of Molecular and Microbiology, George Mason University; Krasnow Institute for Advanced Study, George Mason University

## Abstract

The *Drosophila* peripheral nervous system (PNS) is a powerful model for investigating the complex processes of neuronal development and dendrite morphogenesis at the functional and molecular levels. To aid in these analyses, we have developed a strategy for the isolation of a subclass of PNS neurons called dendritic arborization (da) neurons that have been widely used for studying dendrite morphogenesis^1,2^. These neurons are very difficult to isolate as a pure population, due in part to their extremely low occurrence and their difficult-to-reach location below the tough chitinous larval cuticle. Our newly developed method overcomes these challenges, and is based on a fast and specific cell enrichment using antibody-coated magnetic beads.  For our magnetic bead sorting studies, we have used age-matched third instar larvae expressing a mouse CD8 tagged GFP fusion protein (*UAS-mCD8-GFP*)^3^ under the control of either the class IV dendritic arborization (da) neuron-specific *pickpocket (ppk)-GAL4* driver^4^ or the control of the pan-da neuron-specific GAL4^21-7^ driver^5^. Although this protocol has been optimized for isolating PNS cells which are attached to the inner wall of the larval cuticle, by varying a few parameters, the same protocol could be used to isolate many different cell types attached to the cuticle at larval or pupal stages of development (e.g. epithelia, muscle, oenocytes etc.), or other cell types from larval organs depending upon the GAL4-specific driver expression pattern.  The RNA isolated by this method is of high quality and can be readily used for downstream genomic analyses such as microarray gene expression profiling studies. This approach offers a powerful new tool to perform studies on isolated *Drosophila* dendritic arborization (da) neurons thereby providing novel insights into the molecular mechanisms underlying dendrite morphogenesis.

**Figure:**
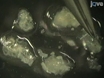


## Protocol

General Comments on Magnetic Bead Sorting of *Drosophila* Peripheral Neurons (Total Timing for the Completion of the Protocol: 2.5-3 hours)

Standard lab procedures for maintaining a clean, RNAse free environment must be observed at all times to prevent RNA degradation.

When the *Drosophila* larval cuticle is dissected and placed in the cell dissociation buffer, the peripheral neurons are one of the last cells to detach from the cuticle. We have exploited this property and designed this protocol to remove most of the non-specific cells from the cuticle such as muscle and fat prior to isolating the da neurons.

With practice, the whole protocol can be successfully completed within approximately 2.5 hours. The preparation of antibody coated beads must be completed before the experiment begins.

### 1. Preparing Magnetic Beads for Binding Cells:

This step must be completed prior to the start of the experiment. The prepared beads can be prepared and stored at 4°C until needed.

1. Wash 100 μl of Dynabeads M-280 Streptavidin coated beads three times in PBS by resuspending it in 500 μl of fresh PBS and pelleting it in a strong magnetic field each time.
2. Finally resuspend the beads directly in 100 μl of undiluted biotinylated rat anti-mouse-CD8a antibody (antibody concentration is 100 μg/ml).
3. Incubate the mixture for 1 hour on ice with occasional mild vortexing to prevent sedimentation. [1 μl of Dynabeads M-280 can bind 0.05-0.10 μg of biotinylated antibody].
4. Wash the bead-antibody mixture three times as described in step 1.1 to remove the excess antibody. The magnetic beads are now coated with antibody and ready to be used. Store the bead-antibody mixture in 100 μl of 1X PBS at 4°C until use.

### 2. Selecting and Washing Larvae: (10-15 minutes)

1. Pick 30-50 age matched third instar larvae and place them in a 1.6ml microfuge tube with 1-1.2 ml of 1X Phosphate Buffered Saline (PBS). (3-5 minutes)
2. Close the tube and vortex it at the maximum setting three times for 1 second each.
3. Using a fire-polished Pasteur pipette discard the supernatant completely. Repeat the wash (2.1) and vortex (2.2) steps 3-4 times until the supernatant is visibly clear of any food particles and debris.
4. Briefly repeat the wash with 1 ml of 70% ethanol and discard the supernatant.
5. Wash the larvae twice with 1 ml of ddH2O and discard the supernatant.
6. Briefly repeat the wash once again with 1 ml of RNase-AWAY and discard the supernatant.
7. Wash the larvae three times in 1 ml of ddH2O to ensure complete removal of RNase-AWAY.

### 3. Dissection: (10-12 minutes)

1. Place 10-12 larvae on the center of a Sylgard coated 35mm Petri plate. Position them slightly away from each other.  [**Critical Step:** Discard any larvae that do not seem to be at the appropriate developmental stage]
2. Cut open the anterior tip of the larvae using a pair of fine dissection scissors for all the larvae.
3. Using a pair of dull Dumont No. 5 forceps invert the larvae inside-out. Insert one forcep inside the larval cuticle all the way to the posterior end. Pinch the tips of the forceps together to grab the posterior end of the cuticle (Figure 1a) (try pressing the cuticle down on the Sylgard surface to make it easier). Using the second pair of forceps, push the larval cuticle inside out.  [**Critical Step:** Try practicing this method a few times before attempting the cell isolation experiment. Try to get the larvae completely inverted, to ensure that all the soft tissues are exposed to the solution for easy dissociation.]
4. After dissecting 3-4 larvae, transfer them immediately to fresh, ice-cold PBS (place the tube on ice) in a 1.6 ml microfuge tube.
5. Repeat steps 3.1 to 3.4 until all the required larvae are collected (30-40 larvae in this protocol). [**Critical Step:** Anticipate 10-20% loss during dissection and dissociation, and plan accordingly.]

### 4. Removal of loosely adherent non-specific cells: (2-3 minutes)

[This step aids the clearing of loosely adherent non-specific tissues such as fat bodies and CNS.]

1. Take the 1.6 ml microfuge tube containing the inverted larval cuticles and replace the supernatant with approximately 700-800 μl of fresh ice-cold PBS.
2. Pulse-vortex the microfuge tube 5 times (3 seconds per pulse) at full speed.
3. Discard the supernatant and replace it with approximately 700-800 μl fresh, ice-cold PBS.
4. Repeat steps 4.2 and 4.3 three times.
5. Resuspend the larval cuticles in 400 μl of fresh, ice-cold PBS

### 5. Dissociating the tissue into a single cell suspension: (18-20 minutes)

[**Critical Step:** Over-dissociation may cause the loss of the cell-surface marker leading to poor cell yield and low cell viability. The larval tissue can be dissociated by either mechanical dissociation (sonication, douncing), enzymatic dissociation (trypsin, collagenase etc.) or a combination of both. As these larval tissues are difficult to dissociate, we found that a combination of both mechanical and enzymatic dissociation yielded the best results.]

1. Add 1.5 μl of 1X Liberase Blendzyme 3 (28 W nsch units/vial) to the larval cuticle suspended in 400 μl of PBS.
2. Vortex the solution 2-3 times for 1 second each at maximum setting (This should remove the loosely adherent cells from the cuticle into the solution).
3. Incubate the solution at room temperature (22-25°C) for 5 minutes. [**Critical Step:** Incubation time greatly affects the final cell sorting efficiency. The recommended incubation time should be sufficient for loosening the tissue. Do not exceed 15 minutes.]
4. Pulse-vortex the tube 20-30 times at maximum settings for 2 seconds per pulse at full speed. This should releases the muscle and other tissue into the solution. Inspect a small sample from the solution under a fluorescent stereo-microscope at each step. (With experience one should be able to determine the level of dissociation by observing the microfuge tube directly under a fluorescence enabled stereo-microscope)
5. Wash the larval cuticles 2-3 times with fresh, ice-cold PBS and finally resuspend them in 500 μl of fresh, ice-cold PBS containing 1% BSA.
6. To avoid the larval cuticle sticking to the glass surface of the 2 ml Kontes tissue grinder and large clearance pestle, pre-coat the tissue grinder and pestle with a 1% BSA in PBS solution and after a brief rinse discard the BSA solution.  Subsequently, using a fire-polished Pasteur pipette, transfer the cuticles from step 5.5 to the BSA-coated tissue grinder. [**Critical Step:** Pre-cool the tissue grinder/pestle by placing it on ice for a few minutes to prevent cell damage/lysis].
7. Dounce the tissue with slow and steady strokes, avoiding frothing (approximately 20-30 strokes). [**Critical Step:** Dounce slowly and steadily, otherwise the cells may lyse.]
8. To assess the cell dissociation levels, wipe the outer wall of the tissue grinder with a clean Kimwipe tissue, and inspect it under a fluorescent stereo-microscope. The neurons should have detached from the cuticle, and can be seen in the solution.  If this is found to be difficult, alternatively pipette out a small sample of the solution, and observe it under a fluorescent stereo-microscope.  A good indication of cell dissociation is the absence of neurons from the larval cuticle. However, if one still observes neurons attached to the cuticle, or observes incompletely dissociated cells, dounce further until the cells achieve a single cell suspension.
9. Triturate the solution 5 times with a fire-polished Pasteur pipette narrowed to approximately 50% of the standard tip diameter, followed by 10 times with a fire-polished Pasteur pipette narrowed to approximately 25% of the standard tip diameter. [**Critical Step:** Forceful trituration may damage the cells.  Monitor the cells between steps, and adjust the procedure accordingly].
10. Filter the solution through a 30 μm cell filter and collect the cell filtrate in a 1.6 ml microfuge tube.  The resulting solution should consist of a single cell suspension and is now ready for magnetic cell sorting.

### 6. Magnetic Bead Cell Sorting: (45 - 75 minutes, dependent on antibody incubation time)

1. Add 15 μl of antibody coated magnetic beads to 500 μl of cell suspension (step 5.10). The remaining antibody conjugated magnetic beads can be stored until needed for subsequent cell isolations.
2. Incubate the cells with antibody coated magnetic beads for 30-60 minutes on ice with occasional hand-mixing. [**Critical Step:**  Incubation at a higher temperature or longer time may result in non-specific antibody binding.]
3. Place the microfuge tube in a strong magnetic field for 2 minutes. All positively selected cells along with unbound beads will separate to the side of the tube.
4. Slowly pipette the supernatant, making sure not to disturb the cell pellet.
5. Wash the cells 3-4 times in fresh, ice-cold PBS to remove any remaining non-specific cells.
6. Resuspend the cells in 30 μl of fresh, ice-cold PBS.
7. To approximate the purity and yield of cells, pipette 5 μl of the cell suspension on the polished surface of a hemocytometer and count all the visible fluorescent cells under a fluorescent stereo-microscope. Also check the amount of non-fluorescent cells and any signs of impurities. Typically the sample will be highly enriched for fluorescent cells.

### 7. RNA Isolation from Magnetic Bead Sorted Cells: (60 – 75 minutes)

1. After counting, pellet the cells in a magnetic field, discard the supernatant and add 20 μl of extraction buffer from the PicoPure™ RNA Isolation Kit (Molecular Devices).  Depending upon cell number one may need to add a higher volume of extraction buffer.
2. Vortex the tube at maximum speed to enable the mixing of cell pellet with extraction buffer.
3. Incubate the tube at 42°C for 30 minutes.
4. To ensure removal of the magnetic beads prior to column purification of the RNA (see step 7.5), the tube is briefly centrifuged at 2,000 (x) g for 2 minutes to pellet the magnetic beads. The tube is then placed in a strong magnetic field to retain the pellet, and the supernatant is transferred to a new microfuge tube.
5. Extract and column purify the RNA according to the PicoPure RNA extraction kit manufacturer’s instructions. DNAse treatment is optional, and can be performed on column during the RNA purification according to the analysis requirement. Finally, elute the bound total RNA in a small volume (11-30 μl) of elution buffer and store at -80°C until ready for use.  If desired a 1 μl aliquot may be used to assess total RNA quality on a Bioanalyzer 2100 (Agilent Technologies, Inc.).

### Representative Results:

Magnetic bead sorting was used to isolate *Drosophila* da neurons (Figure 1). The RNA purified from these isolated da neurons (Figure 2a) was found to be of excellent quality as indicated by the presence of sharp 5.8S, 18S and 28S ribosomal RNA peaks when analyzed on an Agilent 2100 Bioanalyzer (Agilent Technologies, Inc.) (Figure 2b). Beginning with 30-40 third instar larvae we were capable of isolating on average 300-500 class-IV da neurons using the *ppk-GAL4* driver, and 1500-2000 da neurons (Class I,II,III & IV) using the pan-da neuron-specific *GAL4^21-7^* driver.  To assess the neuronal-specific enrichment of our isolated cells we performed quantitative reverse transcription PCR (qRT-PCR) using two neuronal gene-specific markers (*elav* and *futsch*).  These analyses revealed significant fold enrichment of both marker genes indicating a highly specific enrichment for da neurons as compared to flow through using our protocol (Figure 3).  Finally, the isolated RNA from both pan-da neurons and class-IV da neurons was used to perform transcriptional expression profiling on Agilent *Drosophila melanogaster* whole-genome oligo microarrays (4 x 44K) (Figure 4).  These analyses identified numerous previously implicated regulators of da neuron dendrite morphogenesis in addition to a broad spectrum of previously uncharacterized molecules and putative signal transduction pathways that potentially play important functional roles in da neuron development.  Studies designed to assess the potential role(s) of these previously uncharacterized molecules in mediating da neuron development, and specifically dendrite morphogenesis, are presently underway.


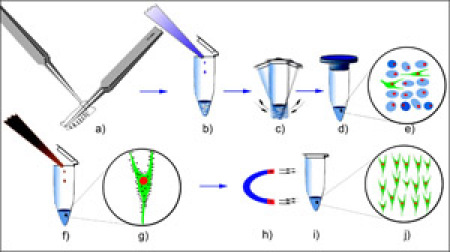
 **Figure 1: **Schematic of magnetic bead sorting of *Drosophila* da neurons.  **(a)** Age matched third instar larvae bearing the da neuron-specific *GAL4,UAS-mCD8-GFP* reporter transgene are dissected by inverting the larval cuticle inside-out, to expose the PNS to dissociation buffer and stored in ice-cold PBS. **(b)** Enzymatic dissociation is carried out by adding Liberase Blendzyme 3 to the solution containing larval cuticle.  **(c)** The larval tissues are further dissociated by a combination of vortexing, trituration and douncing to remove non-specifically labeled tissues such as fat-bodies, gut and CNS. **(d,e)** The cells are then filtered using a 30 μm cell filter. The solution contains a single cell suspension of different cell types including epithelia, muscle and neurons. **(f)** Anti-mouse CD8a-antibody coated Dynabeads M-280 are added to the cell suspension, and incubated on ice for 30-60 minutes.  **(g)** The magnetic beads binds to the da neurons that are expressing a mouse CD8 tagged GFP fusion protein. **(h,i)** The magnetic bead coated cells are separated by placing the solution in a strong magnetic field. The supernatant is discarded, and the cells are washed three times to remove any residual non-specific cells, resulting in **(j)** highly purified populations of da neurons. Please click here to see a larger version of figure 1.


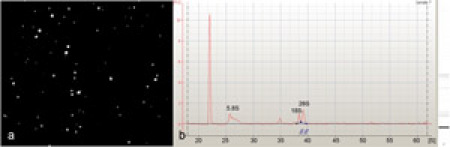
 **Figure 2: (a)** Representative image of positively selected, GFP fluorescent class-IV da neurons isolated by cell dissociation and magnetic bead sorting.  The resulting population of neurons was determined to be highly enriched for class-IV da neurons with little or no contaminating cell impurities. **(b)** An Agilent 2100 Bioanalyzer (Agilent Technologies, Inc.) electropherogram of total RNA isolated from magnetic bead sorted da neurons, showing an excellent total RNA quality as indicated by the presence of 5.8S, 18S, and 28S rRNAs. Please click here to see a larger version of figure 2.


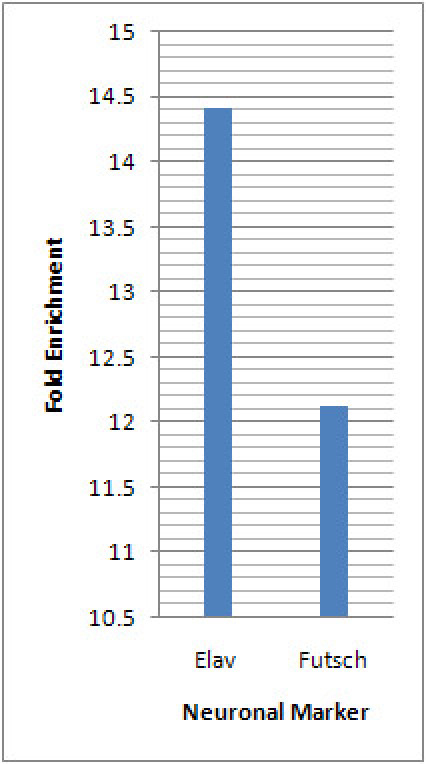
 **Figure 3:** qRT–PCR analysis of neuronal marker gene expression in isolated da neurons (*GAL421-7,UAS-mCD8-GFP*) and the flow through fraction was performed in triplicate. The expression levels of the two neuronal-specific marker genes (elav and futsch) were assessed by qRT–PCR. Values obtained from these analyses were normalized to the endogenous control (*rp49*), and the levels relative to those observed in flow through fraction were calculated using the ΔΔCτ method^6^. Both *elav* and *futsch* were significantly enriched in the isolated da neuron population as compared to the flow through fraction.


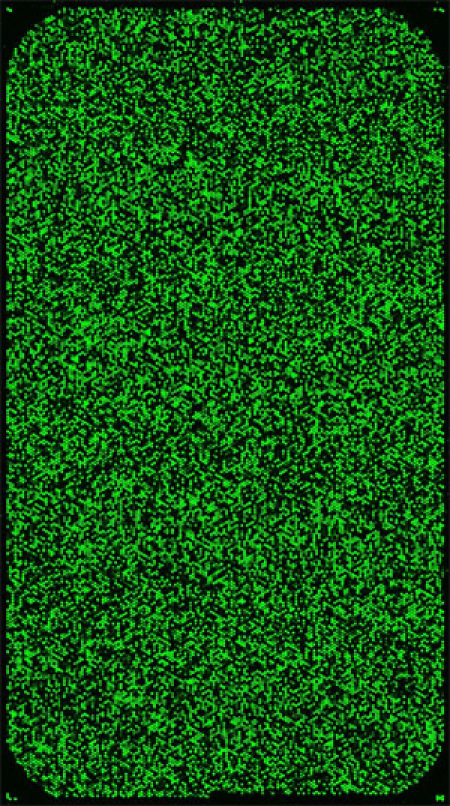
 **Figure 4:** Representative class-IV da neuron-specific Cy3 labeled microarray image file. Shown here is an Agilent Drosophila melanogaster whole-genome oligo microarray (4 x 44K) hybridized with Cy3-labeld total RNA isolated from class-IV da neurons purified by magnetic bead sorting. Please click here to see a larger version of figure 4.

## Discussion

The protocol presented here is optimized for the isolation and purification of peripheral neurons which adhere tightly to the inner surface of the Drosophila third instar larval cuticle using a magnetic bead cell sorting strategy.  While we have used this protocol to specifically isolate Drosophila da neurons, applications of this protocol to the isolation of other cell types that adhere to the cuticle in larval or pupal stages of development (e.g. epithelia, muscle, other peripheral neurons) can be adapted by varying a few parameters and using distinct *GAL4,UAS-mCD8-GFP* reporter transgenes which label the cell type or types of interest.  Moreover, this protocol can be used in both loss-of-function and gain-of-function approaches where a gene of interest may be cloned into a *UAS-mCD8-GFP* transgene that can be coupled with a *GAL4* transgene to direct either gene-specific loss-of-function (*e.g. UAS-RNAi*) or gain-of-function to a cell type of interest.  For example, in the case of a transcription factor one may wish to identify potentially up- or down-regulated genes upon loss-of-function or gain-of-function expression in a cell type of interest.  By isolating total RNA from the purified cell type of interest via this protocol and using this RNA to perform microarray expression profiling it is possible to identify differentially regulated genes that may represent downstream targets of transcriptional regulation that play a role in mediating phenotypic changes within the cell.

For successful cell sorting it is essential to give careful attention to the critical steps highlighted in the above protocol.  Examples of common problem areas that may require some further troubleshooting and optimization, depending upon cell type, include (1) low cell yield and (2) cell clumping during magnetic bead isolation.  In the first case, one may try reducing the concentration of Liberase Blendzyme 3, and compensate by increasing mechanical dissociation via douncing.  In the second case, one may try reducing the magnetic field strength by applying a single or multiple layers of adhesive lab tape over the magnet.
